# Eating Habits during the COVID-19 Pandemic and the Level of Antibodies IgG and FRAP—Experiences of Polish School Staff: A Pilot Study

**DOI:** 10.3390/foods11030408

**Published:** 2022-01-30

**Authors:** Anna Puścion-Jakubik, Ewa Olechno, Katarzyna Socha, Małgorzata Elżbieta Zujko

**Affiliations:** 1 Department of Bromatology, Faculty of Pharmacy with the Division of Laboratory Medicine, Medical University of Białystok, Mickiewicza 2D Street, 15-222 Białystok, Poland; katarzyna.socha@umb.edu.pl; 2 Department of Food Biotechnology, Faculty of Health Science, Medical University of Białystok, Szpitalna 37 Street, 15-295 Białystok, Poland; ewa.olechno@sd.umb.edu.pl (E.O.); malgorzata.zujko@umb.edu.pl (M.E.Z.)

**Keywords:** antioxidants, Poland, lifestyle, diet

## Abstract

The coronavirus 19 (COVID-19) pandemic has brought many changes in terms of lifestyle, education, stress levels, and social contacts. The aim of our research was to evaluate changes in eating habits, physical activity, and selected lifestyle elements in a group of school staff, as well as their immune response to vaccination against COVID-19, and FRAP (ferric reducing antioxidant power) level. In total, 108 primary school teachers and other school staff with integration departments were included in the study. An original survey was conducted with the school staff. Of the study group, 45.4% chose to be vaccinated against COVID-19. In this group, the level of IgG antibodies was assessed, as well as the level of FRAP before vaccination, and after the first and second dose. An original questionnaire was also carried out. A decrease in physical activity and an increase in the time spent in front of the computer have been demonstrated, but a positive observation was a favorable change in most eating habits. After the second dose of vaccination, all subjects achieved the appropriate level of IgG antibodies (above 22 U/mL), with the maximum level recorded in 51%. There was also a significant increase in FRAP levels in the group after the first and second dose of the vaccine compared to the baseline level; an issue that requires further observation.

## 1. Introduction

The COVID-19 pandemic, i.e., acute respiratory distress syndrome, began in December 2019 in the city of Wuhan in central China, and continues to date [1]. On 30 January 2020, the World Health Organization classified COVID-19 as a public health threat of international concern [2]. Severe Acute Respiratory Syndrome Coronavirus 2 (SARS-CoV-2) is a new type of virus from the coronavirus family. Infection occurs through direct or indirect contact with a sick person and their secretions, especially as a result of talking, coughing, and sneezing [1,3]. SARS-CoV-2 infection can be asymptomatic or accompanied by symptoms, including fever, headache and dizziness, runny nose, joint pain, characteristic loss of smell and taste, and disturbed consciousness [1,3,4]. Complications after passing the virus are varied and are still widely studied. People who have had a hard time of COVID-19 may experience shortness of breath and physical and mental weakness after leaving the hospital. It is not clear how long the complications may persist [5]. The pandemic has caused a number of changes in societies around the world in health, social, and economic life [6]. States have taken appropriate measures to minimize the spread of the virus, such as disinfecting hands, rooms, and equipment, as well as making it compulsory to wear masks [7].

The UK was the first Western country to introduce vaccination against SARS-CoV-2 [8]. The World Health Organization (WHO) has officially approved seven types of vaccines. Two contain viral mRNA, three are based on non-replicating viral vectors, and the other two contain inactivated viruses [9]. Some people are concerned about the introduced vaccines, and are withholding a decision to vaccinate [10]. This is due, inter alia, to the lack of confidence in vaccines and the fear of side effects [10,11]. By 21 October 2021, according to WHO, 6,655,399,359 vaccine doses have been administered [12]. It seems that vaccinations can actually have a positive effect on reducing the severity of the disease, which is especially important in the elderly and those with chronic diseases [13]. Vaccinations are designed to stimulate our immune system, to create immune memory, and thus alleviate the course of the disease [14]. According to a 2021 study, the most common symptom reported by 78% of respondents after the first dose of vaccination was pain at the vaccination site. Other symptoms include pain in the extremities (47% of respondents), and fatigue (30%). Malaise, headaches, increased body temperature, and pain in muscles and joints occurred much less frequently [15].

COVID-19 has increased the level of stress in societies around the world. The reasons are, inter alia, uncertainty about work, and the need to quickly adapt to the prevailing conditions [16,17,18]. Increasing the share of stress also translated into a change in eating habits. Studies have found that the pandemic and related quarantine resulted in higher food consumption, which, in turn, led to weight gain (up to 30%) [19]. An increase in the consumption of comfort food such as sweets and fast-food was observed [19,20]. Moreover, a decrease in physical activity was also noted [21,22]. On the other hand, in smokers, the frequency of smoking increased, and in people addicted to alcohol, there was a higher consumption [19]. These changes also adversely affect the body’s resistance by generating oxidative stress. This seems to be particularly important in the prevailing pandemic [23,24]. The diet should include ingredients with antioxidant properties, such as polyphenols, antioxidant vitamins (vitamin C, β-carotene, vitamin E, and vitamin D), as well as minerals (zinc, selenium, copper, and manganese) [24,25]. It has been shown that a high content of these components has a positive effect on the antioxidant potential of the organism [26,27]. In the Prevention with Mediterranean Diet (PREDIMED)study, one-year dietary intervention in the form of the Mediterranean diet significantly increased the FRAP index, which assesses the non-enzymatic antioxidant capacity in the blood by reducing iron ions [28]. Antioxidants are present, among others, in vegetables, fruits, nuts, seeds, herbs, and legumes [29,30,31].

School employees are an important professional group, exposed to contact with many people, unlike, inter alia, administration employees who worked remotely more often. Moreover, these people constitute a model of behavior for their charges; therefore, their behavior may shape the nutritional and health behavior among the young generation.

Due to the fact that the COVID-19 pandemic has caused a number of negative effects, including deterioration in mental and physical health, there is a constant need to investigate this issue. Therefore, the main aim of the study was to assess the eating habits and selected lifestyle elements in a school staff group, and the secondary aim was to assess their immune response to vaccination against COVID-19, and changes in FRAP levels.

## 2. Materials and Methods

### 2.1. Ethical Approval

The study was conducted in accordance with the Helsinki Declaration and Good Clinical Practice, as well as was approved by the Ethics Committee of the Medical University of Bialystok, Poland (approval numbers: APK.002.20.2021, date of approval: 28 January 2021). Informed consent was given by all participants of the study.

### 2.2. Study Design and Participants

In total, 114 school employees residing in the Podlaskie Voivodeship (Bialystok, Poland) were invited to participate in this study. The study participants were employed in a primary school with integration departments, in which disabled children were taught. Therefore, despite the pandemic and school closures, this school worked in a hybrid system. This means that the school worked alternately online and stationary.

**Figure 1:**
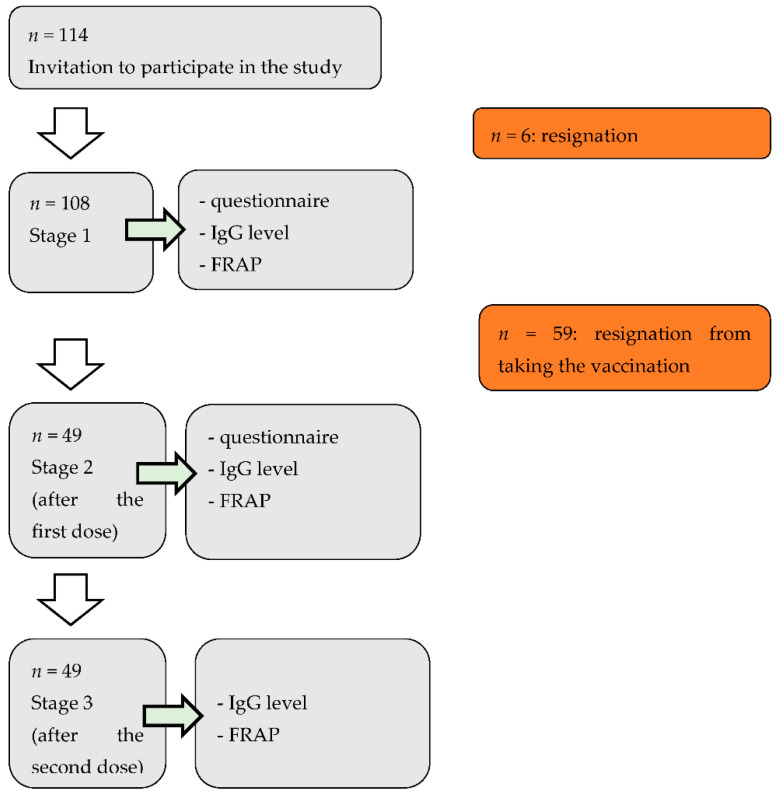
Study scheme. FRAP: ferric reducing antioxidant potential.

The survey was conducted from February to May 2021, and included three periods (before vaccination, 10 and 11 February; two weeks after the first dose of vaccination, 24 March; and two weeks after the second dose of vaccination, 19 May). At the first visit, the participants completed the basic questionnaire (participants personally collected the questionnaires, and had the opportunity to get answers to all questions from the persons conducting the research), and blood was collected for testing (the blood was taken by a professional nurse through a venous puncture). Body weight and height were entered independently by the respondents. Out of 114 respondents, we obtained completed questionnaires from 108 people. The subjects who decided to vaccinate against COVID-19 (45.4%) were invited to the second and third period of the study. Blood samples were taken again during the second and third period of the study, and an additional questionnaire was used. FRAP and antibodies were tested before, and after the first and second dose of vaccination (Figure 1).

The key indicators in our study are: IgG level; FRAP level; and selected anthropometric, lifestyle, and vaccine response aspects. They are characterized below.

### 2.3. Applied Questionaires

The basic questionnaire consisted of three parts: Part 1—questions about gender, age, height, weight, and weight changes during the COVID-19 pandemic, as well as type of work and seniority at school, current form of work (stationary, remotely), and own opinion on the topic of online learning during a pandemic; Part 2—questions about getting COVID-19, symptoms and complications, antibody levels, illness among household members, being in quarantine, chronic diseases, and vaccination against COVID-19; Part 3—questions about changes in eating habits and lifestyle during the pandemic, including questions about experiencing stress, changes in hygiene habits, smoking, physical activity, changes in the consumption of different groups of products, the amount of food consumed per day, time spent in front of the computer, and hours of sleep (Table S1). An additional questionnaire included questions on the post-vaccination aspects: symptoms experienced and their duration (Table S2).

### 2.4. FRAP Assay

The total antioxidant potential of serum was measured spectrophotometrically using the FRAP (ferric reducing antioxidant power) method according to Benzie and Strain [32] on the spectrophotometer UV-Shimadzu (Shimadzu, Kyoto, Japan). This method is based on the reduction of the Fe^3+^ ions in the form of a complex with 2,4,6-Tri(2-pyridyl)-s-triazine (TPTZ) to the Fe^2+^ ions. The TPTZ-Fe^2+^ complex has intense color with a maximum absorption at 593 nm wavelength. The intensity of color is directly proportional to the concentration of Fe^2+^ ions.

### 2.5. Antibodies Assay

The antibodies were measured in microplate reader (Rayto RT-6100C, Guangzhou, China) at 450 nm, using an immunoenzymatic kit for the determination of IgG antibodies against RBD (receptor-binding domain), Spike S1 protein subunit, and SARS-CoV-2 virus (COVID-19) in human serum or plasma (TestLine Clinical Diagnostics s.r.o., Brno, Czech Republic). The RBD specifically binds to the angiotensin-converting enzyme 2 (ACE2) of the host cell. The binding of RBD to ACE2 is highly associated with the formation of neutralizing antibodies.

The interpretation of the antibody level was as follows: lower than 18 U/mL is negative, 18 to 22 U/mL is borderline, and higher than 22 is positive. The maximum level that can be determined with this test is 400 U/mL.

Additionally, the Index of Positivity (IP) was calculated based on the following formula:

$$\mathrm{IP}=\frac{\mathrm{Absorbance}\;\mathrm{of}\;\mathrm{serum},\;\mathrm{plasma}}{\mathrm{Mean}\;\mathrm{absorbance}\;\mathrm{of}\;\mathrm{CUT}\text{-}\mathrm{OFF},}$$

where: CUT-OFF—calibrator, 20 U/mL.

### 2.6. Statystical Analysis

Statistical analyses were performed using Statistica v. 13.3 (StatSoft, TIBCO Software Inc., Palo Alto, CA, USA). The results were considered statistically significant for *p* < 0.05. The Shapiro–Wilk, Kolmogorov–Smirnov, and Lilliefors tests were used to test the normality of the distribution of variables. The Mann–Whitney U test was used to compare groups without a normal distribution. A chi-squared test was used for variables expressed as categories. Correlations were calculated using the Spearman’s test.

## 3. Results

### 3.1. Characteristics of the Group

The first stage of our research involved 108 school employees, including 89 women and 19 men. The mean age of the study group was 46.3 ± 10.5 years, and the mean BMI was over 25 (26.3 ± 4.3 kg/m^2^). The average professional experience of the respondents was 16.6 ± 12.1 years. The results are presented as the number of people (Table 1). 

Among those who reported an increase in their weight, the highest percentage of the studied group (39.0%) reported an increase in body weight by 3–5 kg. The largest percentage of the surveyed school employees conducted classes in grades 0–3 (37.0%) and 4–8 (30.6%). During the completion of the questionnaire, most of the respondents worked stationary (51.4%). The vast majority of respondents assessed that distance learning is worse than traditional education (85.4%) (Table 2).

### 3.2. COVID Infection-Symptoms, Health Background

Of the study group, 21.3% of the respondents tested positive for COVID-19: all patients were female, but we did not show statistical significance (*p* > 0.05). Earlier, before our study, 7.4% of the study group had the level of IgG antibodies determined (Table 3).

Respondents indicated that during COVID-19 infection, they mainly had the following symptoms: smell and taste disorders (14.8%), muscle aches and fatigue (13.9%), fever of 38 °C and above (10.2%), and cough (9.3%) In our study, 13% of respondents indicated that their household tested positive for COVID-19 (Table 3)

The most common reason for quarantine was illness of household members; this reason was indicated by 13.9% of the respondents. About 1/3 of the respondents indicated that they suffer from chronic diseases (34.3%). None of the respondents had been vaccinated against COVID-19, and 56.5% declared their willingness to be vaccinated. Of those declaring that they did not want to be vaccinated, the main reason was that they did not like the type of vaccine offered to healthcare professionals (18.5%) (Table 3).

Among people suffering from COVID-19, 15.7% declared that their health status did not return to the pre-disease state, and the most frequently declared complications were: neurological and psychiatric (7.4%), general (6.5%), and cardiovascular (5.6%).

### 3.3. Lifestyle

Further questions were related to lifestyle changes during the pandemic. As many as 77.8% of respondents declared that they felt stress related to the pandemic: 79.8% of women and 68.4% of men. The main cause of anxiety was concern for their own health and that of their family: this was declared by 81.5% of respondents. Interestingly, as many as 42.6% of school employees indicated that the cause of stress was concern about the level of the teaching of their students. Hygiene habits changed during the pandemic. As many as 86.1% of school employees declared wearing the mask in public places, and 73.1% indicated that they disinfect their hands more often (Table 4).

Only one person indicated that they quit smoking during the pandemic, regular smoking was declared by 7.4% of respondents, and 3.7% assessed that they smoked occasionally (Table 4).

Overall, 61.1% of respondents indicated that their eating habits did not change during the pandemic, and 18.5% of respondents indicated a negative change (Table 4).

It is disturbing to note that the pandemic significantly affected the physical activity of the respondents: as many as 35.2% of the school employees indicated no activity at all during the pandemic (Table 5).

### 3.4. Consumption of Products

We also assessed how eating habits changed during the pandemic. We found significant increases in the consumption of water (21.3%); fruits, vegetables, and salads (20.4%); groats, rice, and cereals (19.4%); tea (19.4%); fish and fish products (16.7%); honey and bee products (15.7%); nuts (11.1%); and eggs (10.2%) (Table 6).

A favorable observation was that the consumption of ready-made dishes for quick preparation at home (23.1%) and alcohol (11.1%) decreased (Table 6).

In addition, we recorded an increase in the number of meals consumed during the day: before the pandemic, only 0.9% said they consumed more than five meals; and during the pandemic, this percentage was as much as 13.0%. The pandemic had a significant impact on the time spent in front of the computer. Before the pandemic, as many as 54.7% indicated that they spent less than 2 h in front of the computer a day. During the pandemic, the highest percentage (31.5%) reported spending 6 to 8 h in front of the computer. The highest percentage of school employees reported sleeping from 7 to 9 h (70.4% vs. 65.7%) (Table 7).

### 3.5. Vaccination

Assessment of the IgG antibody level in the group of school employees before vaccination (*n* = 108) showed that 53.7% of the subjects had low levels of antibodies. In the group of people who were vaccinated in the second stage, an antibody level below 18 U/mL was recorded in 51%. The first dose resulted in a high level of protection (antibody levels above 22 U/mL were recorded in 89.8% of school employees), and after the second dose, this was recorded in 100% of respondents (Table 8). Similar data (percentage of people) were obtained when calculating the percentage of people who responded positively after vaccination (Table 8). This table presents the percentage of people depending on the value of the IP parameter.

An interesting observation was the significant increase in the FRAP level after the first vaccination and after the second vaccination, compared to the baseline level (1484.0 and 1581.0 vs. 1428) (Table 9).

We also saw a significant increase in antibody levels in the first dose and second dose groups compared to pre-vaccination antibody levels (400.0 and 270 vs. 17.8 U/mL) (Table S3).

The preventive vaccinations carried out protected almost the entire studied population of school employees from COVID-19 infection: only one person declared infection after the first dose of the vaccine. The main symptoms in this person were high fever and cough. The people we tested did not have the level of antibodies determined by another laboratory. After vaccination, the main symptoms were: forearm pain (81.6%); muscle aches and fatigue (59.1); and shivering and feeling cold (49.0%). Symptoms in most of the respondents started between 7 and 12 h (59.1% of respondents), and usually disappeared after 24 h (59.2% of respondents) (Table S4).

In the further stage of data analysis, we divided the school staff who had been vaccinated into two groups: one group did not reach the maximum level of antibodies that could be demonstrated by the test, and the other group reached the maximum level of IgG antibodies. We found statistically significant differences only in the case of the antibody level after the first dose of vaccination (140.0 vs. 400, *p* < 0.001), and between the IP index before vaccination (0.635 vs. 1.920, *p* < 0.001) and after the first vaccination (2.670 vs. 4.380, *p* < 0.001). These two groups were very similar in terms of anthropometric parameters (Table S5).

We noticed no differences in the change in body weight between the two groups, as well as in the type of work performed, and thus, a different possibility of contact with potential pathogens (Table S6).

Our research confirmed the link between lower antibody levels and no previous COVID-19 disease. Other factors, such as a positive COVID-19 test among household members, and chronic diseases, had no effect. A disturbing observation is the fact that 24% of respondents with the maximum level of antibodies reported that after infection, they had not yet recovered to their pre-disease state of health (Table S7).

Both groups declared the occurrence of pandemic-related stress to a similar degree, and the main reason was concern for their own health and that of their family (83.3% and 80.0%, respectively). A positive change in eating habits was declared by 25.0% and 20.0% of the respondents, respectively (Table S8).

The frequency of undertaking physical activity was also not related to the achieved antibody level: no statistical significance was shown (Table S9).

People who achieved the antibody level of 400 U/mL indicated, inter alia, an increased consumption of fruit, vegetables, and salads, and honey and bee products; however, we noticed that the change in consumption of the analyzed product categories was not related to the body’s response to vaccination (Table S10).

We noted a difference between the time spent in front of the computer before the pandemic between the two study groups: 62.4% spent less than 2 h a day in front of a computer in the pre-pandemic period, and in the second study group, this was 44.0% (Table S11).

In the group that did not have peak antibody levels after the second dose, one person developed COVID-19 between the first and second doses. Among post-vaccination symptoms, hand pain was more frequent in the group that reached the maximum antibody level (88.0% vs. 75.0%), but these differences were not statistically significant (Table S12).

By analyzing the correlations between the studied parameters, we saw a significant, very high positive correlation between the level of FRAP before vaccination and after the first dose (R = 0.92, *p* < 0.0001), before vaccination and after the second dose (R = 0.93, *p* < 0.0001), and after the first dose and the second dose (R = 0.97, *p* < 0.0001). An interesting, but difficult to explain, observation is the positive correlation between the FRAP level and anthropometric parameters, such as body weight, height, and BMI (Table 10).

## 4. Discussion

Teachers and other school employees are a very important social group exposed to daily contact with a large group of young people. For this reason, they should take special care of their health, as well as apply preventive measures.

Our study had two main goals: to assess teachers’ and other school employees changes in eating and health habits during the COVID-19 pandemic, and to assess their response to immunization against this viral infection.

During the pandemic, changes in the daily functioning of societies around the world were observed [33]. Phenomena such as limiting social meetings, uncertain financial situations, work, and distance learning contributed to an increase in the levels of stress, which, in turn, translated into changes in the generally understood lifestyle [34]. In this study, 39% of respondents saw an increase in body weight from 3 to 5 kg during the pandemic, whereas 47.2% of respondents did not notice a change. Weight gain was also observed in other studies [19,35,36,37,38]. Only 8% of the subjects lost weight in the range of 6–10 kg. Both weight loss and weight gain could be related to stress. Studies have noticed a negative impact of isolation on well-being and eating behaviors [39,40]. It is well known that stress can affect caloric intake in two ways: some people skip meals, whereas some eat more beacause of stress [41]. In a study by Zachary et al. (2020), as many as 52% of respondents increased food consumption in response to stress [42]. A study by Pellegrini et al. (2020) also noted a correlation between weight gain and increased levels of anxiety/depression [43]. The increased level of anxiety during the pandemic also increased the risk of eating disorders [38,44]. Increased food consumption in some studies concerned overweight, obese, and elderly people, which may have already resulted from previous bad eating habits [19,45,46]. In this study, the average BMI value indicated overweight, which, according to the cited studies, may increase the risk of maintaining bad eating habits during the pandemic. Weight gain during COVID-19 may also be associated with decreased activity. In this study, the majority of respondents declared a decrease in physical activity as a result of closed gyms, swimming pools, or other sports-related places. The increase in body weight could therefore be related to the disproportionate consumption of calories to the amount of energy expended. Other studies have found no change, a decrease, as well as an increase in physical activity during the COVID-19 pandemic [37,47,48,49].

Regarding the change in food consumption, there was a statistically significant increase in the consumption of water (23%); fruit and vegetables (22%); tea (21%); groats, rice, and cereals (21%); fish and pocessed fish (18%); honey and bee products (17%); nuts (12%); and eggs (11%). Conversely, there was a decrease in ready-to-eat products (25%) and alcohol (12%) compared to what was consumed before the pandemic. Increased consumption of vegetables and fruits may result from greater care for the supply of essential vitamins for fear of viral infection. Studies by Silva et al. (2021) and Salman et al. (2021) also noted an increase in the consumption of vegetables and fruits during the pandemic [49,50]. It is worth noting that the elderly during the pandemic were characterized by a greater decrease in healthy food consumption than the younger generations [51]. Increased consumption of carbohydrate sources has also been noted in other studies [19,20,45]. The increase in the consumption of fish, honey, bee products, and nuts, as in the case of vegetables, could be due to the desire to ensure immunity. Fish, especially sea fish, are a source of valuable anti-inflammatory omega-3 fatty acids. Their positive effect on the immune functions of the body has been shown [52]. Silva et al. (2021) also observed an increase in fish consumption [49]. Honey, bee products, and nuts are also a source of valuable antioxidants, which could have been important when selecting these products [30,53]. An interesting finding is the increased consumption of eggs, which was also noticed by other researchers [54]. The decline in the consumption of ready-made products seems to be a natural phenomenon, because due to remote work and being locked at home, preparing meals was not as difficult as before the pandemic. Additionally, it may have been associated with a desire to save money in uncertain times. In a study by Molina-Montes et al. (2021), 57.8% of participants reduced the consumption of fast-food dishes, and 52% cooked more often [55]. Other studies also report a decrease in the consumption of ready meals [45,49,54]. A positive change that was observed is the increase in water consumption (23%). Changes in the consumption of tea and alcohol were also significant. Contrary to the study by Błaszczyk-Bębenek et al. (2020) [54], a decrease in alcohol consumption by 12% was observed. Similar conclusions were also drawn by Silva et al. (2021) and Ammar et al. (2020) [49,56]. This may be due to the limitation of social gatherings, as noted by Rehm et al. (2020) [57]. The 2021 review shows the overall increase in alcohol consumption during the COVID-19 pandemic [58]. On the other hand, no changes in coffee consumption were observed, whereas a statistically significant increase in tea consumption was noted, which is in line with the review by Castellana et al. (2021). As the authors emphasize, tea is associated with relaxation, concentration, and being at home [59]. The increase in the consumption of sweets was not statistically significant. Other researchers obtained different results [45,49,54]. An increase in the consumption of sweets was also noted in the review by Gonzalez-Monroy et al. (2021) [58]. Their increased consumption could be associated with an increase in stress accompanying the pandemic [60]. The number of meals per day in this study did not change. In other studies, an increase in food consumption was shown [45,54,61], as well as in snacking [54].

It is well known that a proper diet is necessary to maintain proper immunity. An adequate supply of antioxidant vitamins (vitamin C, β-carotene, vitamin E, and vitamin D), as well as minerals (zinc, selenium, copper, and manganese), polyphenols, and omega-3 fatty acids is particularly important. They regulate the immune system, and thus, reduce the risk of infection. A healthy diet rich in fiber also has a positive effect on the intestinal microbiota, which is extremely important in terms of immunity [62].

Sleep also plays an important role in the context of the body’s immunity. Sleep is important to rapidly combat antigens by cytotoxic NK cells, which peak in the waking period, and to repair damaged body tissues [63]. Sleep time did not change during the pandemic among study participants. However, sleep quality is important and, as noted by Wrigth et al. (2021), may worsen during a pandemic due to the increased level of anxiety [64]. An inadequate amount and quality of sleep also affects eating behavior [65]. The time spent in front of the computer has also increased. Before the pandemic, it was less than 2 h in 59% of the study participants, whereas during the pandemic, only 18% of the participants declared this response. This is mainly due to school activities and remote work. This translates into a decrease in physical activity, which was noticed by other researchers [36].

An interesting observation was that we found a significant increase in FRAP in people who took the first and second doses of the vaccine. The mechanism of this reaction should be clarified in future research. For example, a decrease in FRAP levels has been observed in patients with active Crohn’s disease (0.01 mmol/g of protein) compared to FRAP in patients with inactive disease (0.02 mmol/g of protein) and controls (0.02 mmol/g of protein) [66].

Contreras et al. (2020) conducted a study of the response of animals (cattle) after vaccination against ticks. They measured antioxidant response biomarker parameters, such as: cupric reducing antioxidant capacity (CUPRAC), ferric reducing ability of the plasma (FRAP), trolox equivalent antioxidant capacity (TEAC), total thiol concentrations, and uric acid. The oxidation status was also studied: ferrous oxidation-xylenol orange (FOX), total oxidant status (TOS), advanced oxidation protein products (AOPP), and hydrogen peroxide (H_2_O_2_). A significant decrease in oxidizing markers was observed, with the exception of thiol. The authors concluded that those vaccines that are capable of inducing lower oxidative stress allow the production of higher levels of antibodies [67]. In our study, we observed a high level of FRAP in the group after vaccination 2, which was related to the fact that all people had a positive response and a correspondingly high level of antibodies.

Moreover, we found a positive correlation between FRAP and anthropometric parameters, such as height, weight, and BMI. An increase in FRAP in morbidly obese patients was observed by Choromańska et al. (2020) [68]. This can be explained by an increase in uric acid, which is an endogenous antioxidant. This acid accounts for up to 80% of the total antioxidant potential. In a physiological concentration, it is the most important plasma antioxidant, whereas in higher concentrations, it can generate free radicals (it has pro-inflammatory and pro-oxidative properties). Elevated levels of antioxidant parameters, such as FRAP, may indicate greater ability to remove free radicals, and effective protection against oxidative stress.

In this study, it was found that higher levels of antibodies after vaccination were correlated with higher levels of FRAP, i.e., the body’s ability to reduce iron (III) ions. This is an extremely important observation. However, it is difficult to draw definitive conclusions whether it was a diet rich in antioxidants and, at the same time, increasing the antioxidant status of the body that could have influenced a better response to vaccination. There is a lack of research on this topic. A study on piglets showed promising results, in which it was found that the supplementation of antioxidants with hydrated sodium-calcium aluminosilicates (HASC) increased the level of antibodies after vaccination against the porcine reproductive and respiratory syndrome virus. The supplement consisted of viamine A and E at a dose of 20,000 IU and 200 IU/kg feed, respectively, as well as selenized yeast at a dose of 0.3 mg/kg, and grape seed extract at a dose of 100 mg/kg feed [69].

Changes in eating habits, as well as physical activity, during the COVID-19 pandemic have been noticed by researchers in various parts of the world. It seems that these changes could have been particularly intensified in the initial phase of the pandemic, due to increased stress related to insufficient adaptation to completely new social and economic conditions. It is important to raise public awareness of healthy eating and its impact on the body’s immunity. It appears that the antioxidant status of plasma may have a potential impact on increased immune response to vaccination. This is a new issue, and therefore requires careful research.

The positive correlation observed by us between the FRAP level and anthropometric parameters may indicate the need for further research on the nutritional status of the organism, and the relationship with the parameters of oxidative stress.

There are some limitations to this study. Although it was carried out on the largest group available, further studies should be carried out with a larger number of volunteers. Anthropometric measurements were entered independently by the respondents (due to the pandemic). In further studies, measurements should be performed by a specialist, e.g., a dietitian. Another limitation is the disproportion between the number of women and men. Future research should be gender-balanced. The disproportion in our study reflects the actual gender distribution of school workers.

## 5. Conclusions

The period of the COVID-19 pandemic contributed to a decrease in physical activity among primary school teachers and other school employees, as well as to an increase in the amount of time spent in front of the computer. As this is a conscious and educated group, a compensation for this could be a change in eating habits, including increased consumption of vegetables, fruits, salads, honey and bee products, nuts, fish and processed fish, eggs, groats, rice, cereals, tea, and water; and reduced consumption of ready-made dishes for quick preparation at home, and alcohol. The protective vaccination against COVID-19 contributed to a significant increase in the level of IgG antibodies. There has also been a significant increase in FRAP, but this issue requires further investigation on the link and determination of whether a higher level is a cause or effect.

## Figures and Tables

**Table 1:** Characteristics of the study group (*n* = 108).

Parameter	*n*	Av. ± SD	Med.	Min.–Max	Q1–Q3
Gender (*n*, W/M)	89/19	-	-	-	-
Age (years)	108	46.3 ± 10.5	48.5	24.0–70.0	39.5–54.0
Height (m)	108	168.0 ± 6.6	168.0	153.0–190.0	163.0–170.5
Body weight (kg)	108	74.0 ± 13.5	70.0	55.0–115.0	63.0–83.0
BMI (kg/m^2^)	108	26.3 ± 4.3	25.6	19.3–41.7	23.0–29.1
Work experience (years)	108	16.6 ± 12.1	17.5	0.5–51.0	4.5–20.5

Av.—average, M—men, Max—maximum, Med.—median, Min.—minimum, Q1–Q3—quartile 1–quartile 3, SD—standard deviation, W—women.

**Table 2:** Baseline characteristic of study groups (*n* = 108).

Parameter	Total (*n* = 108)	Women (*n* = 89)	Men (*n* = 19)
	*n* (%)	*n* (%)	*n* (%)
Change in weight during a pandemic			
No change	51 (47.2)	41 (46.1)	10 (52.5)
It was increased in the range of 3–5 kg	42 (39.0)	36 (40.4)	6 (31.6)
It was increased in the range above 10 kg	5 (4.6)	4 (4.5)	1 (5.3)
It was reduced in the range of 3–5 kg	1 (0.9)	0 (0.0)	1 (5.3)
It was reduced in the range of 6–10 kg	8 (7.4)	7 (7.9)	1 (5.3)
It was reduced in the range above 10 kg	1 (0.9)	1 (1.1)	0 (0.0)
Type of work performed at school (multiple choice question)			
Teacher in grades 0–3	40 (37.0)	38 (42.7)	2 (10.5)
Teacher in grades 4–8	33 (30.6)	23 (25.8)	10 (52.6)
School administration	10 (9.3)	8 (9.0)	2 (10.5)
School service	29 (26.9)	24 (27.0)	5 (26.4)
How do you currently work?			
Stationary	55 (51.4)	48 (53.9)	7 (36.8)
Remotely	21 (18.7)	13 (14.6)	8 (42.1)
Stationary and remotely	32 (29.9)	28 (31.5)	4 (21.1)
How do you rate remote learning during a pandemic?			
Comparable to traditional teaching	16 (14.6)	14 (15.7)	2 (10.5)
Worse than traditional education	92 (85.4)	75 (84.3)	17 (89.5)

**Table 3:** Experiences with COVID-19 among study group (*n* = 108).

Parameter	Total (*n* = 108)	Women (*n* = 89)	Men (*n* = 19)
	*n* (%)	*n* (%)	*n* (%)
Have you been tested positive for COVID-19?			
Yes	23 (21.3)	23 (25.8)	0 (0.0)
No	85 (78.7)	66 (74.2)	19 (100.0)
Have you had a COVID-19 antibody test performed?			
Yes	8 (7.4)	8 (9.0)	0 (0.0)
No	100 (92.6)	81 (91.0)	19 (100.0)
If you have had COVID-19, please mark the symptoms accompanying the disease (multiple choice question)			
Fever of 38 °C and above	11 (10.2)	10 (11.2)	1 (5.3)
Cough	10 (9.3)	9 (10.1)	1 (5.3)
Diarrhea	3 (2.8)	3 (3.4)	0 (0.0)
Nausea	4 (3.7)	4 (4.5)	0 (0.0)
Vomiting	1 (0.9)	1 (1.1)	0 (0.0)
Smell and taste disorders	16 (14.8)	15 (16.9)	0 (0.0)
Conjunctivitis	1 (0.9)	1 (1.1)	0 (0.0)
Difficulty breathing, difficulty drawing air	7 (6.5)	7 (7.9)	0 (0.0)
Muscle aches, fatigue	15 (13.9)	14 (15.7)	1 (5.3)
Other symptoms	7 (6.5)	7 (7.9)	0 (0.0)
Have any of your household members had a positive COVID-19 test?			
Yes	14 (13.0)	8 (9.0)	6 (31.6)
No	94 (87.0)	81 (91.0)	13 (68.4)
Were you in quarantine because of COVID-19?			
Yes	36 (33.3)	29 (32.6)	7 (36.8)
No	72 (66.7)	60 (67.4)	12 (63.2)
For what reason were you in quarantine? (38 answers)			
Own disease	6 (5.6)	6 (6.7)	0 (0.0)
Household disease	15 (13.9)	11 (12.4)	4 (21.1)
Co-worker disease	10 (9.3)	7 (7.9)	3 (15.8)
Return from abroad	2 (1.9)	2 (2.2)	0 (0.0)
Another	5 (4.6)	5 (5.6)	0 (0.0)
Do you suffer from chronic diseases?			
Yes	37 (34.3)	34 (38.2)	3 (15.8)
No	71 (65.7)	55 (61.8)	16 (84.2)
Have you been vaccinated against COVID-19?			
No	109 (100.0)	89 (100.0)	19 (100.0)
Yes	0 (0.0)	0 (0.0)	0 (0.0)
Would you report your willingness to be vaccinated against COVID-19 if it was possible?			
Yes	61 (56.5)	47 (52.8)	14 (73.7)
No	47 (43.5)	42 (47.2)	5 (26.3)
If not, why not? (30 answers)			
I don’t believe vaccination is effective	3 (2.8)	2 (2.2)	1 (5.3)
I do not like the type of vaccine offered to the education staff	20 (18.5)	17 (19.1)	3 (15.8)
Other	7 (6.5)	7 (7.9)	0 (0.0)
If you have had COVID-19, do you think that your current health has returned to its pre-disease state? (23 answers)			
Yes	6 (5.6)	6 (6.7)	0 (0.0)
No	17 (15.7)	14 (15.7)	3 (15.8)
If you have suffered from COVID-19, what complications do you experience after the illness? (35 answers)			
General	7 (6.5)	7 (7.9)	0 (0.0)
From the respiratory system	5 (4.6)	5 (5.6)	0 (0.0)
From the cardiovascular system	6 (5.6)	6 (6.7)	0 (0.0)
Neurological and psychiatric	8 (7.4)	8 (9.0)	0 (0.0)
From the gastrointestinal tract	1 (0.9)	1 (1.1)	0 (0.0)
From the motor organ	3 (2.8)	3 (3.4)	0 (0.0)
From the sensory organs and the throat	5 (4.6)	4 (4.5)	1 (5.3)

COVID-19—coronavirus disease.

**Table 4:** Lifestyle changes during a pandemic (*n* = 108).

Parameter	Total (*n* = 108)	Women (*n* = 89)	Men (*n* = 19)
	*n* (%)	*n* (%)	*n* (%)
Do you feel stress related to the pandemic?			
Yes	84 (77.8)	71 (79.8)	13 (68.4)
No	24 (22.2)	18 (20.2)	6 (31.6)
What is the stress experienced during a pandemic related to? (multiple choice question)			
Concern for own and family’s health	88 (81.5)	73 (82.0)	15 (78.9)
Limited social life	42 (38.9)	36 (40.4)	6 (31.6)
Care for job stability and earnings	31 (28.7)	26 (29.2)	5 (26.3)
Online learning and limited access to computer hardware	18 (16.7)	17 (19.1)	1 (5.3)
Concern for the level of the teaching of their students	46 (42.6)	40 (44.9)	6 (31.6)
Other	2 (1.9)	2 (2.2)	0 (0.0)
How have your hygiene habits changed during the pandemic? (multiple choice question)			
They have not changed	15 (13.9)	13 (14.6)	2 (10.5)
I wash my hands more often	72 (66.7)	59 (66.3)	13 (68.4)
I disinfect my hands more often	79 (73.1)	66 (74.2)	13 (68.4)
I wear the mask in public places	93 (86.1)	76 (85.4)	15 (78.9)
Other	3 (2.8)	2 (2.2)	0 (0.0)
Do you smoke cigarettes?			
Yes, regularly	8 (7.4)	3 (3.4)	5 (26.3)
Yes, occasionally	4 (3.7)	3 (3.4)	1 (5.3)
No	95 (88.0)	83 (93.2)	12 (63.1)
I have smoked, but quit during the pandemic	1 (0.9)	0 (0.0)	1 (5.3)
How do you evaluate the change in eating habits during the pandemic?			
Positive change	22 (20.4)	20 (22.5)	2 (10.5)
Negative change	20 (18.5)	13 (14.6)	6 (31.6)
No change	66 (61.1)	55 (62.9)	11 (57.9)

**Table 5:** Physical activity before and during a pandemic (*n* = 108).

Physical Activity	Before the Pandemic *	During a Pandemic
	*n* (%)	*n* (%)
Lack of physical activity	23 (21.3)	38 (35.2)
1–2 times a week, minimum 30 min	43 (39.9)	42 (38.9)
3–5 times a week, minimum 30 min	25 (23.1)	17 (15.7)
More than 5 times a week, minimum 30 min	17 (15.7)	11 (10.2)

* *p* < 0.05—statistically significant differences between groups.

**Table 6:** Changes in product consumption during a pandemic (*n* = 108).

Product	Increase in Consumption	Decrease in Consumption
	*n* (%)	*n* (%)
Fruits, vegetables, salads	22 (20.4) **	3 (2.8)
Honey and bee products	17 (15.7) ***	1 (0.9)
Nuts	12 (11.1) *	3 (2.8)
Milk and dairy products	12 (11.1)	5 (4.6)
Meat and meat products	12 (11.1)	17 (15.7)
Fish and processed fish	18 (16.7) **	3 (2.8)
Eggs	11 (10.2) *	2 (1.9)
Bread	7 (6.5)	14 (13.0)
Groats, rice, cereals	21 (19.4) **	7 (6.5)
Flour preparations (pies, pancakes, rolls, cookies)	18 (16.7)	13 (12.0)
Sweets	21 (19.4)	15 (13.9)
Ready-made dishes for quick preparation at home	7 (6.5)	25 (23.1) **
Coffee	16 (14.8)	13 (12.0)
Tea	21 (19.4) **	6 (5.6)
Juices	10 (9.3)	12 (11.1)
Water	23 (21.3) ***	4 (3.7)
Alcohol	3 (2.8)	12 (11.1) *

* *p* < 0.05, ** *p* < 0.01, *** *p* < 0.001—statistically significant differences between groups.

**Table 7:** Changes in eating during a pandemic (*n* = 108).

Parameter	Before the Pandemic	During a Pandemic
	*n* (%)	*n* (%)
Number of meals during the day		
1–2 meals	11 (10.2)	10 (9.2)
3–5 meals	96 (88.9)	84 (77.8)
over 5 meals	1 (0.9)	14 (13.0)
Time spent in front of the computer		
less than 2 h a day	59 (54.7) ***	18 (16.7)
2–3 h a day	31 (28.7)	15 (13.9)
4–5 h a day	10 (9.2)	21 (19.4)
6–8 h a day	3 (2.8)	34 (31.5)
more than 8 h a day	5 (4.6)	20 (18.5)
Hours of sleep per day		
6 h or less	31 (28.7)	33 (30.6)
7–9 h	76 (70.4)	71 (65.7)
10 or more hours	1 (0.9)	4 (3.7)

*** *p* < 0.01—statistically significant differences between women and men.

**Table 8:** Percentage of people by the IgG and by the rate of positive reaction after vaccination.

Parameter	Before Vaccination (*n* = 108)	Before Vaccination (*n* = 49)	After First Vaccination (*n* = 49)	After Second Vaccination (*n* = 49)
	*n* (%)	*n* (%)	*n* (%)	*n* (%)
IG				
Under 18 U/mL	58 (53.7)	25 (51.0)	5 (10.2)	0 (0.0)
18–22 U/mL	8 (7.4)	4 (8.2)	0 (0.0)	0 (0.0)
Above 22 U/mL	42 (38.9)	20 (40.8)	44 (89.8)	49 (100.0)
Index of Positivity				
Under 0.9	58 (53.7)	25 (51.0)	5 (10.2)	0 (0.0)
0.9–1.1	8 (7.4)	4 (8.2)	0 (0.0)	0 (0.0)
Above 1.1	42 (38.9)	20 (40.8)	44 (89.8)	49 (100.0)

**Table 9:** FRAP level in the study group (*n* = 49).

Parameter	Av. ± SD	Med. (Q1–Q3)	*p*
FRAP—before vaccination (A)	1453.3 ± 292.2	1428.0 (1271.0–1599.0)	*p* _A/B_ < 0.0001
FRAP—after 1 dose (B)	1539.7 ± 285.0	1484.0 (1346.0–1721.0)	*p* _A/C_ < 0.0001
FRAP—after 2 doses (C)	1613.1 ± 294.4	1581.0 (1423.0–1787.0)	*p* _B/C_ < 0.0001

Av.—average, FRAP—ferric reducing antioxidant power, Med.—median, Q1–Q3—quartile 1–quartile 3, SD—standard deviation.

**Table 10:** Correlations between the selected parameters in the case of people who achieved the maximum level of IgG antibodies, and those with a lower level.

Group	Parameter 1	Parameter 2	R, *p*
IgG level below 400 (*n* = 24)	FRAP before vaccination	FRAP after first dose	0.92, 0.0001
	FRAP before vaccination	FRAP after second dose	0.93, 0.0001
	FRAP after first dose	FRAP after second dose	0.97, 0.0001
	IgG before vaccination	IgG after first dose	0.56, 0.0042
	IgG before vaccination	IgG after second dose	0.55, 0.0053
	FRAP after second dose	Growth	0.41, 0.0482
IgG level 400 (*n* = 25)	FRAP before vaccination	FRAP after first dose	0.76, 0.0001
	FRAP before vaccination	FRAP after second dose	0.78, 0.0001
	FRAP before vaccination	BMI	0.61, 0.0013
	FRAP after first dose	FRAP after second dose	0.98, 0.0001
	FRAP after first dose	BMI	0.68, 0.0002
	FRAP after second dose	BMI	0.70, 0.0001

## Data Availability

Detailed data is available from the authors.

## Supplementary Materials

The following are available online at https://www.mdpi.com/article/10.3390/foods11030408/s1. Table S1: A survey that was carried out in the first stage of the study. Table S2: A survey that was carried out in the second stage of the study. Table S3: Level of IgG in the study group (*n* = 49). Table S4. Respondents’ reaction to vaccination with the first dose (*n* = 49). Table S5: Comparison of selected parameters between people who achieved the maximum level of IgG antibodies (400 U/mL) and the lower level (*n* = 49). Table S6: Comparison of selected aspects of lifestyle between people who achieved the maximum level of IgG antibodies and the lower level (*n* = 49). Table S7: Comparison of selected aspects of symptoms, quarantine and opinions on vaccinations during the COVID-19 pandemic between people who achieved the maximum level of IgG antibodies (400 U/mL) and the lower level (*n* = 49). Table S8: Comparison of selected aspects of well-being and habits during the COVID-19 pandemic between people who achieved the maximum level of IgG antibodies (400 U/mL) and the lower level (*n* = 49). Table S9: Comparison of physical activity during the COVID-19 pandemic between people who achieved the maximum level of IgG antibodies (400 U/mL) and the lower level (*n* = 49). Table S10: Comparison of changing eating habits between people who achieved the maximum level of IgG antibodies (400 U/mL) and the lower level (*n* = 49). Table S11: Comparison of changing eating habits between people who achieved the maximum level of IgG antibodies (400 U/mL) and the lower level (*n* = 49). Table S12: Post-vaccination information with 1 dose - comparison between people who achieved the maximum level of IgG antibodies and the lower level (*n* = 49).
