# Supplementary material for: Eating Habits during the COVID-19 Pandemic and the Level of Antibodies IgG and FRAP—Experiences of Polish School Staff: A Pilot Study
Source: Foods. 2022 Jan 30;11(3):408. doi: 10.3390/foods11030408 (PMC8834520; doi:10.3390/foods11030408)
Supplement: Supplementary file 1 [file foods-11-00408-s001.zip › foods-1558363-supplementary.pdf]

Supplementary Material

Eating habits during the COVID-19 pandemic and the level of antibodies IgG and FRAP - experiences of Polish school staff: A pilot study

Anna Puścion-Jakubik [1,*,#], Ewa Olechno [2,#], Katarzyna Socha [1] and Małgorzata Elżbieta Zujko [2]

[1] Department of Bromatology, Faculty of Pharmacy with the Division of Laboratory Medicine,
Medical University of Białystok, Mickiewicza 2D Street, 15-222 Białystok, Poland; katarzyna.socha@umb.edu.pl (K.S.)

[2] Department of Food Biotechnology, Faculty of Health Science, Medical University of Białystok, Szpitalna 37 Street, 15-295 Białystok, Poland; ewa.olechno@sd.umb.edu.pl (E.O.); malgorzata.zujko@umb.edu.pl (M.E.Z.)

# contributed equally

Table S1. A survey that was carried out in the first stage of the study.

Number …

Date of completing the survey …

E-mail address or telephone number (for sending or forwarding test results) …

Survey questionnaire

Research topic: Qualitative and quantitative assessment of IgG antibodies against COVID-19 in a group of primary school workers.

I. General information about the patient

Question	Answer
**I. General information about the patient**	
1. Gender	Female/male
2. Age	…
3. Height (cm)	…
4. Current body weight (kg)	…
5. During the pandemic, did your body weight:	It remained at the same level<br>It was increased in the range of 3-5 kg<br>It was increased in the range of 6-10 kg<br>It was increased in the range above 10 kg<br>It was reduced in the range of 3-5 kg<br>It decreased in the range of 6-10 kg<br>It was reduced in the range above 10 kg<br>Another ...
6. Work experience at school	…
7. Type of work at school:	Teacher of grades 0-3<br>Teacher in grades 4-8<br>School administration<br>School service<br>A different kind of work (what?)
8. How do you currently work:	Stationary<br>Remotely<br>Stationary and remotely

9. How do you rate remote learning during a pandemic?	Comparable to traditional teaching<br>Worse than traditional education

II. COVID-19 Information

1. 1. Have you had a COVID-19 test positive?<br>If so, please provide approximate date when you received a positive COVID-19 test	Yes<br>Not
2. Have you been tested for antibodies against COVID-19?<br>If so, please provide approximate date when the antibody test will be performed<br>What was the test result?	Yes<br>Not
3. If you have had COVID-19, please tick the symptoms accompanying the disease:	a) Fever of 38 C and above<br>Yes<br>No<br>b) Cough<br>Yes<br>No<br>c) Diarrhea<br>Yes<br>No<br>d) Nausea<br>Yes<br>No<br>e) Vomiting<br>Yes<br>No<br>f) Disturbances in smell and taste<br>Yes<br>No<br>g) Conjunctivitis<br>Yes<br>No<br>h) Difficulty breathing, shortness of breath, difficulty breathing<br>Yes<br>No<br>i) Muscle aches, fatigue<br>Yes<br>No<br>j) Other symptoms<br>Yes<br>No<br>If so, please list which ...
4. Has any of your household members had a positive COVID-19 test?	Yes<br>No
5. Were you in quarantine for COVID-19?	Yes<br>Not
If so, for what reason	Own disease<br>Household disease<br>Co-workers' disease<br>Return from abroad<br>Other (what?)
6. Do you suffer from chronic diseases?	Yes<br>No
If so, please list which ones?	…
7. Have you been vaccinated against COVID-19?	Yes

	No
8. Would you report your willingness to be vaccinated against COVOD-19, if it was possible?	Yes<br>No
If not, why not:	I don't believe in the effectiveness of the vaccinations<br>I do not like the type of vaccine offered to educators<br>Another ...
9. If you have suffered from COVID-19, do you think that your current health has returned to its pre-disease state?	Yes<br>No
10. If you have suffered from COVID-19, what are the complications after the disease you are currently experiencing (please mark and underline the appropriate one):	general (fever, pain, fatigue)<br>on the part of the respiratory system (cough, feeling short of breath)<br>on the part of the cardiovascular system (pressure and pain in the chest, palpitations)<br>neurological and psychiatric (disturbance of concentration and memory, headaches, sleep disorders, dizziness, depression, anxiety)<br>gastrointestinal (abdominal pain, nausea, diarrhea, appetite disorders)<br>on the part of the musculoskeletal system (muscle pain, joint pain)<br>sensory organ and throat (earache, tinnitus, smell and taste disturbances, sore throat)<br>other

III. Information on lifestyle changes during a pandemic

1. Do you experience any stress related to the pandemic?	Yes<br>No
2. What is the stress associated with a pandemic (you can choose more than 1 answer):	Care for your and your family's health<br>Limited social life<br>Concern for job stability and earnings<br>Remote learning and limited access to computer hardware<br>Concern for the level of teaching of their students and children<br>Other (what?)
3. How have your hygiene habits changed during the pandemic? (you can choose more than 1 answer)	They have not changed<br>I wash my hands more<br>I disinfect my hands more often<br>I wear my mask in public places<br>Other (what?)
4. Do you smoke cigarettes?	Yes, regularly for ... years<br>Occasionally, for ... years<br>Not<br>I have smoked, but quit during the pandemic
5. How did your physical activity change during the pandemic (please mark with a cross in the appropriate boxes)	

Physical activity	Before the pandemic	During the pandemic
Lack of physical activity		
1-2 times a week, minimum 30 minutes		
3-5 times a week, minimum 30 minutes		
More than 5 times a week, minimum 30 minutes		

6. How have your eating habits changed during the pandemic? (please mark with a cross in the appropriate boxes)

Consumption of food products	No change	Increase in consumption	Decrease in consumption
Fruit and vegetables, salads			
Honey and bee products			
Peanuts			
Milk and dairy products			
Meat and meat products			
Fish and processed fish			
Eggs			
Bread			
Groats, rice, cereals			
Flour preparations (pies, pancakes, rolls, cookies)			
Sweets			
Ready-made dishes for quick preparation at home			

7. How do you assess the change in eating habits during the pandemic?

Positive change in eating habits
Negative change in eating habits
No change

8. The frequency of consumption of meals (please mark with a cross in the appropriate fields):

Number of meals during the day	Before the pandemic	During a pandemic
1-2 meals		
3-5 meals		
over 5 meals		

9. How has your time spent in front of the computer changed during the pandemic? (please mark with a cross in the appropriate boxes)

Time spent in front of the computer	Before the pandemic	During a pandemic
Less than 2 hours a day		
2-3 hours a day		
4-5 hours a day		
6-8 hours		
more than 8 hours a day		

10. How has the number of hours of sleep changed during the pandemic? (please mark with a cross in the appropriate boxes)

Hours of sleep	Before the pandemic	During a pandemic
6 hours or less		
7-9 hours		
10 or more hours		

Table S2. A survey that was carried out in the second stage of the study.

Number …
Date of completing the survey …
Vaccination date
E-mail address (preferably school) or telephone number

Post First Dose Antibody Questionnaire - Part 2

Question	Answer
1. Have you suffered from COVID-19 (confirmed by a test) in the period from the first antibody test to the present day?	Yes, I was sick in the period between the first antibody test and vaccination<br>Yes, I was ill after vaccination<br>No, I was not ill during this period
2. If so, what were the symptoms of the disease?	a) Fever of 38 C and above<br>Yes<br>No<br>b) Cough<br>Yes<br>No<br>c) Diarrhea<br>Yes<br>No<br>d) Nausea<br>Yes<br>No<br>e) Vomiting<br>Yes<br>No<br>f) Disturbances in smell and taste<br>Yes<br>No<br>g) Conjunctivitis<br>Yes<br>No<br>h) Difficulty breathing, shortness of breath, difficulty breathing<br>Yes<br>No<br>i) Muscle aches, fatigue<br>Yes<br>No<br>j) Other symptoms:<br>Yes<br>No<br>If so, please list which ones …
3. Did you perform additional antibody tests in the period between the first antibody test and today?	If so, please provide the result …<br>and the date of the test …
4. Did you have any worrying symptoms after taking the first dose?	a) Hand pain<br>Yes<br>No<br>b) Fever of 38 C and above<br>Yes<br>No<br>c) shivering and feeling cold<br>Yes<br>No<br>d) Flu-like symptoms<br>Yes<br>No<br>e) Headache

	Yes<br>No
	f) Nausea<br>Yes<br>No
	g) Vomiting<br>Yes<br>No
	h) Muscle aches, fatigue<br>Yes<br>No
	i) Other symptoms<br>Yes<br>No<br>If so, please list which ...
5. How many hours after receiving the vaccine did the first disturbing symptoms appear?	…
6. How long did these symptoms last?	24 hours<br>48 hours<br>other period of time how?

Table S3. Level of IgG in the study group (n = 49).

Parameter	Av.±SD	Med. (Q1-Q3)	p
Antibody level – before vaccination (A)	77.3±108.4	17.8 (10.5-72.1)	$p_{A/B} < 0.0001$
Antibody level – after 1 dose (B)	262.2±146.5	270.0 (175.0-400.0)	$p_{A/C} < 0.0001$
Antibody level – after 2 dose (C)	346.2±81.4	400.0 (345.0-400.0)	$p_{A/C} < 0.0001$

Table S4. Respondents' reaction to vaccination with the first dose (n = 49).

Parameter	Total (n = 49)	
	n (%)	
Did you suffer from COVID-19 (confirmed by the test) in the period from the first antibody test to the present day?		
Yes, I was sick in the period between the first antibody test and vaccination	0 (0.0)	
Yes, I was ill after vaccination	1 (2.0)	
No, I did not / I did not get sick during this period	48 (98.0)	
If so, what were the symptoms of the disease? (multiple choice question)	**Yes**	**No**
Fever of 38 C and above	1 (2.0)	48 (98.0)
Cough	1 (2.0)	48 (98.0)
Diarrhea	0 (0.0)	49 (100.0)
Nausea	0 (0.0)	49 (100.0)
Vomiting	0 (0.0)	49 (100.0)
Smell and taste disorders	0 (0.0)	49 (100.0)
Conjunctivitis	0 (0.0)	49 (100.0)
Difficulty breathing, difficulty drawing air	0 (0.0)	49 (100.0)
Muscle aches, fatigue	0 (0.0)	49 (100.0)
Other symptoms	0 (0.0)	49 (100.0)
Did you perform additional antibody tests in the period between the first antibody test and today?		
yes	0 (0.0)	
no	49 (100.0)	
Did you have any worrying symptoms after taking the first dose of the vaccine? (multiple choice question)	Yes	No
forearm pain	40 (81.6)	9 (18.4)
fever of 38C and above	22 (44.9)	27 (55.1)
shivering and feeling cold	24 (49.0)	25 (51.0)
flu-like symptoms	21 (42.9)	28 (57.1)
headache	22 (44.9)	27 (55.1)
nausea	2 (4.1)	47 (95.9)
vomiting	1 (2.0)	48 (98.0)
muscle aches, fatigue	29 (59.1)	20 (40.9)
other symptoms	7 (14.3)	40 (85.7)
How many hours after receiving the vaccine did the first alarming symptoms appear?		

6 hours and less	10 (20.4)
7 – 12 hours	29 (59.1)
13 – 18 hours	4 (8.2)
19 – 24 hours	2 (4.1)
lack	4 (8.2)
How long did the symptoms last?	
24 hours	29 (59.2)
48 hours	11 (22.4)
different period of time	5 (10.2)
lack	4 (8.2)

Table S5. Comparison of selected parameters between people who achieved the maximum level of IgG antibodies (400 U/mL) and the lower level (n = 49).

**Parameter**	**Antibodies Levels Below 400 (*n* = 24)**	**Antibodies at the Level 400 (*n* = 25)**
	***n* (%)**	***n* (%)**
Gender (n, W/M)	19/5	21/4
Age (years)	47.3±9.2<br>(28.0-64.0)<br>50.0<br>(43.0-52.5)	47.8±9.8<br>(25.0-59.0)<br>49.0<br>(45.0-56.0)
Growth (cm)	168.2±6.2<br>(160.0-181.0)<br>167.0<br>(163.5-171.0)	165.8±6.5<br>(156.0-184.0)<br>165.0<br>(162.0-170.0)
Body weight (kg)	74.3±11.1<br>(60.0-96.0)<br>70.5<br>(64.0-85.0)	75.0±15.7<br>(55.0-115.0)<br>72.0<br>(62.0-85.0)
BMI (kg/m^2)	26.3±4.1<br>(21.0-36.6)<br>25.3<br>(23.2-28.3)	27.2±5.3<br>(21.0-41.7)<br>25.6<br>(23.1-30.5)
IgG level before vaccination	21.8±36.2<br>(5.7-180.7)<br>12.6<br>(9.0-18.0)	130.5±127.3<br>(5.7-323.6)<br>60.9***<br>(17.8-272.1)
IgG level after first vaccination	140.4±111.2<br>(10.0-290.0)<br>140.0<br>(27.5-255.0)	379.2±44.5<br>(270.0-400.0)<br>400.0***<br>(400.0-400.0)
FRAP level before vaccination	1484.2±327.7<br>(1068.0-2506.0)<br>1381.0<br>(1302.5-1599.0)	1424.1±256.9<br>(881.0-1912.0)<br>1443.0<br>(1256.0-1599.0)
FRAP level after first vaccination	1538.0±336.7<br>(1006.0-2412.0)<br>1473.5<br>(1299.5-1668.0)	1541.2±231.9<br>(1181.0-1959.0)<br>1506.0<br>(1356.0-1721.0)
FRAP level after second vaccination	1633.7±3247.6<br>(1186.0-2511.0)<br>1546.0<br>(1417.5-1759.5)	1593.4±263.8<br>(1124.0-2245.0)<br>1581.0<br>(1124.0-2245.0)
IP before vaccination	0.771±0.596<br>(0.290-3.030)<br>0.635<br>(0.450-0.900)	2.318±1.466<br>(0.290-4.420)<br>1.920***<br>(0.890-3.720)
IP after first vaccination	2.438±1.340<br>(0.400-4.140)<br>2.670<br>(1.230-3.725)	4.380±0.186<br>(3.670-4.380)<br>4.380***<br>(4.380-4.380)
Work experience (years)	20.1±10.1<br>(3.0-40.0)	20.8±12.0<br>(0.5-36.0)

	20.0 (15.0-30.0)	24.0 (10.0-30.0)

*** $p < 0.001$ - statistically significant differences between groups.

Table S6. Comparison of selected aspects of lifestyle between people who achieved the maximum level of IgG antibodies and the lower level ($n = 49$).

Questions	Antibodies levels below 400 ($n = 24$)	Antibody at the Level 400 ($n = 25$)
	***n* (%)**	***n* (%)**
**Change in weight during a pandemic**		
No change	11 (45.8)	13 (52.0)
It was increased in the range of 3-5 kg	8 (33.3)	8 (32.0)
It was increased in the range above 10 kg	1 (4.2)	2 (8.0)
It was reduces in the range of 3-5 kg	0 (0.0)	0 (0.0)
It was reduced in the range of 6-10 kg	4 (16.7)	2 (8.0)
It was reduced in the range above 10 kg	0 (0.0)	0 (0.0)
**Type of work performed at school (multiple choice question)**		
Teacher in grades 0-3	9 (37.5)	7 (28.0)
Teacher in grades 4-8	13 (54.2)	11 (60.0)
School administration	2 (8.3)	2 (8.0)
School service	3 (12.5)	6 (24.0)
**How do you currently work**		
Stationary	8 (33.3)	14 (56.0)
Remotely	9 (37.5)	7 (28.0)
Stationary and remotely	7 (29.2)	4 (16.7)
**How do you rate remote learning during a pandemic?**		
Comparable to traditional teaching	4 (16.7)	3 (12.0)
Worse than traditional education	20 (83.3)	22 (88.0)

Table S7. Comparison of selected aspects of symptoms, quarantine and opinions on vaccinations during the COVID-19 pandemic between people who achieved the maximum level of IgG antibodies (400 U/mL) and the lower level ($n = 49$).

Questions	Antibodies levels below 400 ($n = 24$)	Antibodies at the Level 400 ($n = 25$)
	***n* (%)**	***n* (%)**
**Have you been tested positive for COVID-19?**		
Yes	2 (8.3)	10 (40.0)
No	22 (91.7)*	15 (60.0)
**Have you had a COVID-19 antibody test performed?**		
Yes	2 (8.3)	2 (8.0)
No	22 (91.7)	23 (92.0)
**If you have had COVID-19, please mark the symptoms accompanying the disease (multiple choice question)**		
Fever of 38 °C and above	1 (4.2)	6 (24.0)
Cough	0 (0.0)	5 (20.0)
Diarrhea	0 (0.0)	2 (8.0)
Nausea	1 (4.2)	2 (8.0)
Vomiting	1 (4.2)	0 (0.0)
Smell and taste disorders	1 (4.2)	8 (32.0)
Conjunctivitis	0 (0.0)	0 (0.0)
Difficulty breathing, difficulty drawing air	0 (0.0)	5 (20.0)
Muscle aches, fatigue	1 (4.2)	7 (28.0)
Other symptoms	1 (4.2)	4 (16.0)
**Has any of your household members had a positive COVID-19 test?**		
Yes	4 (16.7)	5 (20.0)
No	20 (83.3)	20 (80.0)
**Were you in quarantine because of COVID-19?**		
Yes	6 (25.0)	10 (40.0)
No	18 (75.0)	15 (60.0)
**For what reason were you in quarantine? (14 answers)**		
Own disease	1 (4.2)	4 (16.0)

Household disease	2 (8.3)	2 (8.0)
Co-workers disease	1 (4.2)	2 (8.0)
Return from abroad	0 (0.0)	1 (4.0)
Another	0 (0.0)	1 (4.0)
**Do you suffer from chronic diseases?**		
Yes	8 (33.3)	5 (20.0)
No	16 (66.7)	20 (80.0)
**Have you been vaccinated against COVID-19?**		
No	24 (100.0)	25 (100.0)
Yes	0 (0.0)	0 (0.0)
**Would you report your willingness to be vaccinated against COVID-19 if it was possible?**		
Yes	24 (100.0)	23 (92.0)
No	0 (0.0)	2 (8.0)
**If not, why not? (2 answers)**		
I don't believe vaccination is effective	0 (0.0)	1 (4.0)
I do not like the type of vaccine offered to the education staff	0 (0.0)	1 (4.0)
Other	0 (0.0)	0 (0.0)
**If you have had COVID-19, do you think that your current health has returned to its pre-disease state? (10 answers)**		
Yes	1 (4.2)	2 (8.0)
No	1 (4.2)	6 (24.0)
**If you have suffered from COVID-19, what complications do you experience after the illness? (21 answers)**		
general	0 (0.0)	4 (16.0)
from the respiratory system	0 (0.0)	4 (16.0)
from the cardiovascular system	1 (4.2)	3 (12.0)
neurological and psychiatric	0 (0.0)	5 (20.0)
from the gastrointestinal tract	0 (0.0)	0 (0.0)
from the motor organ	0 (0.0)	2 (8.0)
from the sensory organs and the throat	1 (4.2)	0 (0.0)
other	0 (0.0)	1 (4.0)

Table S8. Comparison of selected aspects of well-being and habits during the COVID-19 pandemic between people who achieved the maximum level of IgG antibodies (400 U/mL) and the lower level (*n* = 49).

Questions	Antibodies levels below 400 (*n* = 24)	Antibodies at the Level 400 (*n* = 25)
	***n* (%)**	***n* (%)**
**Do you feel stress related to the pandemic?**		
Yes	21 (87.5)	18 (72.0)
No	3 (12.5)	7 (28.0)
**What is the stress experienced during a pandemic related to? (multiple choice question)**		
concern for own and family's health	20 (83.3)	20 (80.0)
limited social life	11 (45.8)	11 (44.0)
care for job stability and earnings	4 (16.7)	5 (20.0)
on-line learning and limited access to computer hardware	3 (12.5)	4 (16.0)
concern for the level of teaching of their students	8 (33.3)	11 (44.0)
other	0 (0.0)	0 (0.0)
**How have your hygiene habits changed during the pandemic? (multiple choice question)**		
they have not changed	2 (8.3)	3 (12.0)
I wash my hands more often	17 (70.8)	17 (68.0)
I disinfect my hands more often	19 (79.2)	22 (88.0)
I wear the mask in public places	20 (83.3)	24 (96.0)
other	1 (4.2)	0 (0.0)
**Do you smoke cigarettes?**		
Yes, regularly	4 (4.2)	0 (0.0)
Yes, occasionally	2 (8.3)	2 (8.0)
Not	18 (87.5)	23 (92.0)
I have smoked, but quit during the pandemic	0 (0.0)	0 (0.0)
**How do you evaluate the change in eating habits during the pandemic?**		
positive change	6 (25.0)	5 (20.0)
negative change	5 (20.8)	7 (28.0)
no change	13 (54.2)	13 (52.0)

Table S9. Comparison of physical activity during the COVID-19 pandemic between people who achieved the maximum level of IgG antibodies (400 U/mL) and the lower level (*n* = 49).

**Physical activity**	**Antibodies levels below 400 (*n* = 24)**		**Antibodies at the Level 400 (*n* = 25)**	
	**Before pandemic**	**During a pandemic**	**Before pandemic**	**During a pandemic**
	***n* (%)**	***n* (%)**	***n* (%)**	***n* (%)**
Lack of physical activity	3 (12.5)	8 (33.3)	5 (20.0)	11 (44.0)
1-2 times a week, minimum 30 minutes	12 (50.0)	9 (37.5)	11 (44.0)	9 (36.0)
3-5 times a week, minimum 30 minutes	5 (20.8)	4 (16.7)	6 (24.0)	4 (16.0)
more than 5 times a week, minimum 30 minutes	4 (16.7)	3 (12.5)	3 (12.0)	1 (4.0)

Table S10. Comparison of changing eating habits between people who achieved the maximum level of IgG antibodies (400 U/mL) and the lower level (*n* = 49).

**Questions**	**Antibodies levels below 400 (*n* = 24)**		**Antibodies at the Level 400 (*n* = 25)**	
	**Increase in consumption**	**Decrease in consumption**	**Increase in consumption**	**Decrease in consumption**
	***n* (%)**	***n* (%)**	***n* (%)**	***n* (%)**
Fruit and vegetables, salads,	2 (8.3)	1 (4.2)	5 (20.0)	1 (4.0)
Honey and bee products	2 (8.3)	1 (4.2)	4 (16.0)	0 (0.0)
Nuts	6 (25.0)	1 (4.2)	3 (12.0)	0 (0.0)
Milk and dairy products	3 (12.5)	0 (0.0)	2 (8.0)	2 (8.0)
Meat and meat products	2 (8.3)	5 (20.8)	3 (12.0)	4 (16.0)
Fish and processed fish	5 (20.8)	1 (4.2)	4 (16.0)	1 (4.0)
Eggs	1 (4.2)	0 (0.0)	3 (12.0)	1 (4.0)
Bread	1 (4.2)	0 (0.0)	4 (16.0)	2 (8.0)
Groats, rice, cereals	5 (20.8)	2 (8.3)	8 (32.0)	0 (0.0)
Flour preparations (pies, pancakes, rolls, cookies)	6 (25.0)	4 (16.7)	7 (28.0)	3 (12.0)
Sweets	5 (20.8)	4 (16.7)	5 (20.0)	2 (8.0)
Ready-made dishes for quick preparation at home	0 (0.0)	8 (33.3)	2 (8.0)	8 (32.0)
Coffee	3 (12.5)	2 (8.3)	4 (16.0)	3 (12.0)
Tea	4 (16.7)	0 (0.0)	7 (28.0)	1 (4.0)
Juices	4 (16.7)	4 (16.7)	1 (4.0)	2 (8.0)
Water	6 (25.0)	2 (8.3)	5 (20.0)	0 (0.0)
Alcohol	3 (12.5)	8 (33.3)	0 (0.0)	1 (4.0)

Table S11. Comparison of changing eating habits between people who achieved the maximum level of IgG antibodies (400 U/mL) and the lower level (*n* = 49).

**Parameter**	**Antibodies levels below 400 (*n* = 24)**		**Antibodies at the Level 400 (*n* = 25)**	
	**Before pandemic *n* (%)**	**During a pandemic *n* (%)**	**Before pandemic *n* (%)**	**During a pandemic *n* (%)**
**Number of meals during the day**				
1-2 meals	4 (16.7)	3 (12.5)	3 (12.0)	1 (4.0)
3-5 meals	19 (79.1)	17 (70.8)	22 (88.0)	17 (68.0)
over 5 meals	1 (4.2)	4 (16.7)	0 (0.0)	7 (28.0)
**Time spent in front of the computer**				
less than 2 hours a day	15 (62.4)***	2 (8.2)	11 (44.0)	4 (16.0)
2-3 hours a day	6 (25.0)	1 (4.2)	8 (32.0)	3 (12.0)
4-5 hours a day	1 (4.2)	7 (29.2)	4 (16.0)	4 (16.0)
6-8 hours a day	1 (4.2)	7 (29.2)	1 (4.0)	11 (44.0)
more than 8 hours a day	1 (4.2)	7 (29.2)	1 (4.0)	3 (12.0)
**Hours of sleep per day**				
6 hours or less	9 (37.5)	10 (41.7)	6 (24.0)	4 (16.0)
7-9 hours	15 (62.5)	12 (50.0)	18 (72.0)	19 (76.0)
10 or more hours	0 (0.0)	2 (8.3)	1 (4.0)	2 (8.0)

*** $p < 0.001$ - statistically significant differences between groups.

Table S12. Post-vaccination information with 1 dose - comparison between people who achieved the maximum level of IgG antibodies and the lower level (*n* = 49).

Parameter	Antibodies levels below 400 (*n* = 24)	Antibodies at the Level 400 (*n* = 25)
	n (%)	*n* (%)
**Did you suffer from COVID-19 (confirmed by the test) in the period from the first antibody test to the present day?**		
Yes, I was sick in the period between the first antibody test and vaccination	0 (0.0)	0 (0.0)
Yes, I was ill after vaccination	1 (4.2)	0 (0.0)
No, I did not / I did not get sick during this period	23 (95.8)	25 (100.0)
**If so, what were the symptoms of the disease? (2 answers)**		
Fever of 38 °C and above	1 (4.2)	0 (0.0)
Cough	1 (4.2)	0 (0.0)
Diarrhea	0 (0.0)	0 (0.0)
Nausea	0 (0.0)	0 (0.0)
Vomiting	0 (0.0)	0 (0.0)
Smell and taste disorders	0 (0.0)	0 (0.0)
Conjunctivitis	0 (0.0)	0 (0.0)
Difficulty breathing, difficulty drawing air	0 (0.0)	0 (0.0)
Muscle aches, fatigue	0 (0.0)	0 (0.0)
Other symptoms	0 (0.0)	0 (0.0)
**Did you perform additional antibody tests in the period between the first antibody test and today?**		
Yes	0 (0.0)	0 (0.0)
No	24 (100.0)	25 (100.0)
**Did you have any worrying symptoms after taking the first dose of the vaccine? (multiple choice question)**		
forearm pain	18 (75.0)	22 (88.0)
fever of 38 °C and above	11 (45.8)	11 (44.0)
shivering and feeling cold	12 (50.0)	12 (48.0)
flu-like symptoms	11 (45.8)	10 (40.0)
Headache	12 (50.0)	10 (40.0)
Nausea	2 (8.3)	0 (0.0)
Vomiting	1 (4.2)	0 (0.0)
muscle aches, fatigue	14 (58.3)	15 (60.0)
other symptoms	5 (20.8)	2 (8.0)
**How many hours after receiving the vaccine did the first alarming symptoms appear?**		
6 hours and less	3 (12.5)	7 (28.0)
7 – 12 hours	19 (79.2)	10 (40.0)
13 – 18 hours	0 (0.0)	4 (16.0)
19 – 24 hours	0 (0.0)	2 (8.0)
Lack	2 (8.3)	2 (8.0)
**How long did the symptoms last?**		
24 hours	18 (75.0)	10 (40.0)
48 hours	1 (4.2)	10 (40.0)
different period of time	3 (12.5)	3 (12.0)
Lack	2 (8.3)	2 (8.0)
